# Drug-Class Specific Impact of Antivirals on the Reproductive Capacity of HIV

**DOI:** 10.1371/journal.pcbi.1000720

**Published:** 2010-03-26

**Authors:** Max von Kleist, Stephan Menz, Wilhelm Huisinga

**Affiliations:** 1 Hamilton Institute, Computational Physiology Group, National University of Ireland Maynooth, Kildare, Ireland; 2 Department of Mathematics and Computer Science, Freie Universität Berlin, Berlin, Germany; ETH Zurich, Switzerland

## Abstract

Predictive markers linking drug efficacy to clinical outcome are a key component in the drug discovery and development process. In HIV infection, two different measures, viral load decay and phenotypic assays, are used to assess drug efficacy *in vivo* and *in vitro*. For the newly introduced class of integrase inhibitors, a huge discrepancy between these two measures of efficacy was observed. Hence, a thorough understanding of the relation between these two measures of drug efficacy is imperative for guiding future drug discovery and development activities in HIV. In this article, we developed a novel viral dynamics model, which allows for a mechanistic integration of the mode of action of all approved drugs and drugs in late clinical trials. Subsequently, we established a link between *in vivo* and *in vitro* measures of drug efficacy, and extract important determinants of drug efficacy *in vivo*. The analysis is based on a new quantity—the reproductive capacity—that represents in mathematical terms the *in vivo* analog of the read-out of a phenotypic assay. Our results suggest a drug-class specific impact of antivirals on the total amount of viral replication. Moreover, we showed that the (drug-)target half life, dominated by immune-system related clearance processes, is a key characteristic that affects both the emergence of resistance as well as the *in vitro*–*in vivo* correlation of efficacy measures in HIV treatment. We found that protease- and maturation inhibitors, due to their target half-life, decrease the total amount of viral replication and the emergence of resistance most efficiently.

## Author Summary

To guide drug discovery and development, measures of drug efficacy that are linked to clinical outcome are of key importance. In HIV treatment, decay of plasma viral load is typically used as an *in vivo* measure of drug efficacy, whereas phenotypic assays are used to assess drug efficacy *in vitro*. The recent development of novel HIV drugs resulted in a huge discrepancy between viral load decay and *in vitro* predictions of drug efficacy. We used a mathematical modelling approach to resolve this discrepancy by introducing a new quantity, the reproductive capacity, that allows a transfer of the *in vitro* drug efficacy measure into the *in vivo* context, enabling a direct comparison. We developed a novel model of viral dynamics that incorporates the mechanism of action of all established and novel antivirals. Based on the model, we analyzed the ability of the viral infection to replicate under different drug treatments, and estimated class-specific times until virological failure. We conclude that the half life of the targeted viral stage is an important class-specific attribute that impacts on the overall success of a drug *in vivo*. Our findings have direct implication for the drug discovery and development process.

## Introduction

Since 1996, human immunodeficiency virus (HIV) infection is treated with a combination therapy, known as highly active anti-retroviral therapy (HAART) [1],[2], which has substantially improved the clinical management of HIV [3]. Despite the success of HAART, eradication of HIV can currently not be achieved [4],[5], most likely due to the persistence of virus in very long lived, latently infected cells [6],[7]. For HIV-infected individuals, life-long therapy is therefore required to prevent progression to the acquired immunodeficiency syndrome (AIDS) and death.

During therapy, plasma viral load (HIV RNA per mL blood plasma) is recommended by the National Institute of Health as a marker of therapy success [8], whereas measurement of the CD4 cell count is the most important clinical marker of disease progression [9]. The *in vivo* potency of novel antivirals is usually assessed by viral load decline in small clinical trials of monotherapy, e.g., [10],[11], and later evaluated utilizing the novel agent in combination with an optimized background therapy, e.g., [12]. The *in vitro* potency of antivirals is typically assessed by using phenotypic/single-round infectivity assays [13]–[16], which measure the number of offspring after one round of virus replication.

Investigation of novel drug targets for the treatment of HIV infection resulted in the development of new drug classes. In 2003 and 2007, the fusion inhibitor (FI) enfuvirtide [17], the CCR5-antagonist maraviroc [18] and the integrase inhibitor raltegravir [19] were approved for the treatment of HIV infection. Many more drugs are in late clinical development [20]. With the introduction of new drug classes, in particular integrase inhibitors, a huge discrepancy between the efficacy measured *in vitro*, using phenotypic/single-round infectivity assays, and *in vivo*, using viral load decline, was observed [14],[21]. Although integrase inhibitors cause a steep initial decline of plasma viral load [21]–[26], the *in vitro* efficacy is amongst the lowest [14].

Mathematical modelling of viral dynamics has lead to many insights into the pathogenesis and treatment of HIV. It is a valuable tool to interpret the time course of virological markers (e.g. viral load) during HIV treatment [27]–[31] and contributes much to our current understanding of the *in vivo* dynamics of HIV. Sedaghat et al. [32],[33] used a mathematical modelling approach to analyze the rapid decay of plasma viral load after application of integrase inhibitors. They infer that this characteristic viral decay is a result of the inhibited stage within the viral life cycle rather than superior *in vivo* potency.

Consequently, viral load decay may be misleading for assessing the potency of integrase inhibitors (and other novel inhibitors) in comparison to existing drug classes. However, an alternative, more appropriate measure of drug efficacy, which allows to directly compare drugs from different drug classes is still missing.

The objectives of this article are (i) to develop a novel, generic measure of drug potency that facilitates comparison across different drug classes; (ii) to develop a novel mathematical model of the viral replication cycle that incorporates the action of established and novel drugs in a *mechanistic* way; and (iii) to analyze determinants of drug efficacy critical for drug discovery and development. The proposed measure of drug efficacy, termed reproductive capacity, extends the established *in vivo* marker, plasma viral load, by incorporating additional infectious viral stages, and the *in vitro* phenotypic/single-round infectivity assays by taking into account host specific defense mechanisms. This enables us to understand the observed discrepancies between *in vitro* and *in vivo* efficacy for integrase inhibitors, and to elucidate and quantify the role of immune-system related clearance mechanisms in drug action. The results presented herein are of particular value to categorize different molecular targets in the HIV life cycle and are expected to be of significance for guiding future HIV drug discovery and development.

## Results

### Development of a detailed model of viral life cycle and action of anti-retroviral drugs

**Figure 1:**
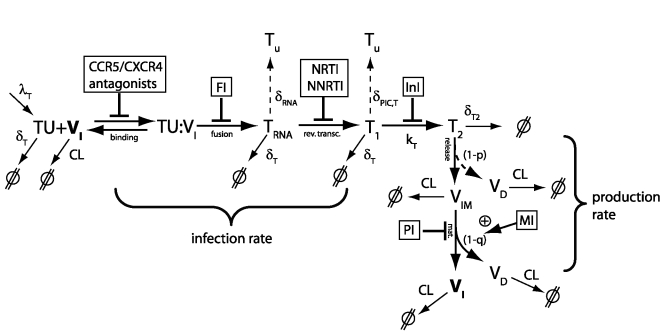
Detailed structural model of the viral life cycle and the mechanisms of action of different anti-retroviral drug classes.

We derived a detailed virus-target cell interaction model as depicted in Fig. 1. The model incorporates the mechanisms of action of all currently approved drugs and some drugs in late clinical development.

Target cells are produced by the immune system with some constant rate 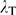. An infectious virus 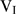 reversibly binds (with effective rate constants 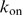 and 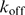) to a target cell 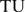, forming a complex 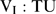. After binding, the virus irreversibly fuses (with rate constant 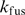) with the target cell and the viral capsid containing the viral genomic RNA is released; this state is denoted by 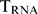. During reverse transcription (with effective rate constant 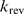), genomic viral RNA is irreversibly transformed into a more stable DNA. Viral DNA and viral proteins form the pre-integration complex (PIC), denoted by 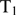. In the next step, viral DNA of the PIC is irreversibly integrated into the DNA of the target cell (with rate constant 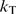), forming the provirus 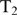. After integration, the infected cell cannot return to an uninfected stage. From the proviral DNA, viral proteins are amplified and new viruses are released (with effective rate constant 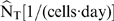). Only a given percentage 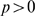 of the released viruses are correctly assembled immature viruses 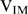, while the remaining percentage 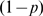 are defective virions 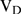 that might e.g. lack the (*gag-pol*-polyprotein contained) enzymes. During the final step, the viral protease, which is packed into the correctly assembled, immature virions 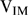, is responsible for the maturation of the virus. The maturation of HIV virions has been shown to be dependent on the highly ordered cascade of cleavages, governed by differences in the inherent processing rates at each cleavage site [34],[35]. We assume that a fraction 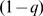 of the released virus matures abnormally, contributing to the pool of defective virions 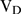. Successful maturation eventually leads to new infectious virus particles 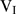 (with rate constant 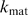 and probability 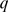).

Depending on the stage of the life cycle, the host organism has different abilities to clear the virus. It was assumed that infectious, immature and defective virions 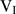, 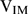, and 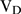, respectively, are cleared with rate constant 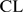 by the host. The uninfected target cells 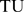, the 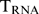 stage and the early infected stage 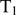 are assumed to be cleared with rate constant 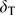, since none of these stages express viral proteins, while the virus-producing late infected cell 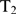 is assumed to be cleared with rate constant 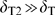. In addition to cell death, the target cell may fend-off the viral infection by degrading the viral RNA or parts of the PIC, rendering the cell uninfected. RNA is very unstable with a half life ranging from seconds to a maximum of two hours [36],[37]. Therefore, through degradation or, e.g., by hypermutation through APOBEC3G [38], the viral RNA can be cleared with rate constant 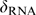. The cell might also destroy essential components of the PIC (with rate constant 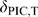) to clear the virus.

The system of ordinary differential equations (ODEs) describing the rate of change of the different viral species and target cells in the detailed model (depicted in Fig. 1) is given in Supplementary Text S1, Eqs. (S1)–(S8). As typically done in kinetic studies, complex aspects of the viral dynamics are subsumed by ‘lumped’ parameters in the model. For instance, the rate constant of the reverse transcription 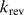 contains all the steps necessary to transform the viral RNA into a double stranded DNA. The mechanisms of action of the seven drug classes are based on interfering with the viral life cycle at different stages. We assumed that the effect of a drug on the targeted process is specified by some parameter 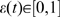, i.e.,
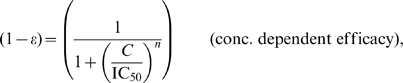
(1)assuming some underlying averaged drug concentration 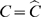, see [39], some fifty percent inhibitory concentration 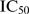, and some drug specific Hill coefficient 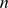, see [14]. For the purpose of the study, this rough approximation is sufficient, however, it is possible to also use time-varying drug concentration 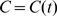 resulting from some pharmacokinetic model, or to use more mechanistic effects models [40],[41].

The actions of the different drug classes within the viral life cycle are shown in Fig. 1. CCR5 antagonists inhibit the association of HIV with the CCR5 receptor in CCR5-tropic virus. They thus affect the association constant 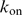. Fusion inhibitors (FI) inhibit the process of HIV fusion, affecting 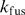. Activated nucleoside reverse transcriptase inhibitors (NRTI) compete with endogenous deoxynucleoside triphosphates for prolongation of the growing DNA chain, while non-nucleoside reverse transcriptase inhibitors (NNRTI) allosterically inhibit the function of the reverse transcriptase. The effects of both drug classes result in a reduced rate at which the RNA is reversely transcribed into DNA. Integration inhibitors affect the integration of viral DNA into the host genome catalytically [42]–[45]. In the proposed model, this alters the transition rate constant 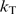 from early infected cells 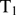 to the late infected cells 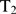. Protease inhibitors (PI) bind to the catalytic pocket of the viral protease enzyme, which is responsible for the processing of the viral precursor polyproteins and thus the maturation of viral particles. In the proposed model (Fig. 1), PIs therefore inhibit maturation by decreasing the maturation constant 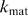. Maturation inhibitors (MI) bind to the substrate of the viral protease (*Gag*-polyprotein) [46] at a specific site. This binding perturbs the ordered sequence of cleavages that is necessary for proper maturation [47],[48], resulting in defective virus morphology [49]. In the proposed model (Fig. 1), MIs therefore decrease the probability 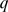 that immature virus matures normally, increasing the proportion of abnormally matured, defective viruses 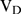.

### Impact of antiviral drugs on relative abundance of infectious viral stages

**Figure 2:**
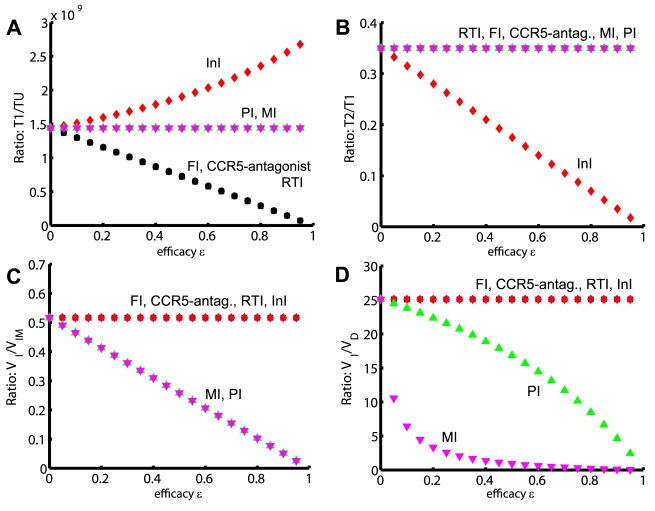
Mechanistic effects of drug classes on viral infective compartments. Ratios are affected through treatment with different drug classes. Predictions are based on the detailed model (see Fig. 1) and mechanistic effect 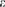 varying from 0–1. Chosen parameter values: 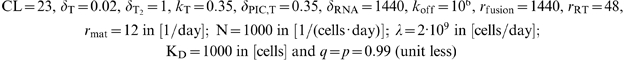

We used the detailed virus-target cell interaction model to predict the effect of the different drug classes on the distinct stages of the viral life cycle. In order to enable a direct comparison between the different drug classes, we artificially eliminated the feedback by keeping the uninfected target cell 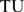 and the infective virions 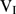 that ‘enter’ the infection cycle constant (the two leftmost species in Fig. 1), resulting in ‘downstream’ quasi-steady state numbers 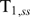, 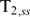, 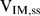, 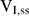, and 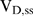. For a given drug class and inhibition of the targeted molecular process 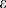, the effect of the drug on the life cycle was quantified by the four ratios
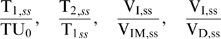
(2)as shown in Fig. 2. As expected, the drugs perturb the ratios of viral states that encompass their site of action within the viral life cycle. In the present example, all states that lie downstream of the drugs' target site are affected, while the states that lie upstream are usually not affected. The exception are InIs, which increase the abundance of the preceding stage 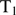 (Fig. 2A), while decreasing the number of the subsequent infectious stage 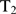 (Fig. 2B). Interestingly, the effect on the ratios is not always a linear function of drug efficacy. PIs and MIs also show a different behavior (Fig. 2D): PIs affect the ratio of infectious-to-defective virions by decreasing the maturation rate 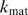, which lowers the number of infective virions 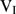, but also lowers the number of virions that mature abnormally (contributing to 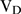). MIs increase the proportion of virus that matures abnormally and decrease the proportion of virus that matures normally, thus decreasing 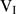 and increasing 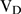, without affecting 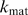.

### Development of a simplified two stage virus dynamics model

The detailed model (Fig. 1) contains parameters that are difficult to measure and currently not available. We therefore reduced the detailed model based on reasonable quasi-steady state assumptions to obtain a simplified model of virus-target cell interaction dynamics that is parameterizable in terms of established and validated parameter values (see Supplementary Text S1). In particular, we have eliminated the intermediate stages of the cell-virus complex 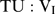, the infected cells prior to reverse transcription 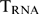 and the immature virus 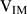 in the original model (Fig. 1). As a consequence, we derived a lumped infection rate constant 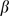, which describes the infection of a susceptible cell towards the stage, where the viral RNA has been successfully transformed into DNA. We also derived a virus clearance 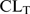 that is associated with the loss of virus during the intermediate stages before reverse transcription and the release rate constant of infectious virus 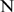.

The infection rate constant is given by
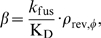
(3)where 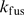 denotes the fusion rate constant, 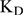 the dissociation constant of the virus-target cell complex, and 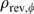 denotes the probability that reverse transcription is successfully completed (see Supplementary Text S1). The lumped virus clearance (loss of virus by, e.g., genome destruction) in the intermediate stages is given by the parameter
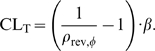
(4)The number of released, infectious viruses is given by
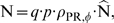
(5)where 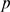 and 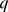 are the probabilities that the released virus is correctly assembled and matures normally, and 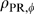 is the probability that the released virus matures before being cleared by the immune system (see Supplementary Text S1). The lumped model can be parameterized in terms of six unknown parameters (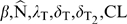), which equals the number of estimated parameters using standard models [28]. For the remaining parameters, we have provided values from the literature (see Supplementary Text S1).

**Figure 3:**
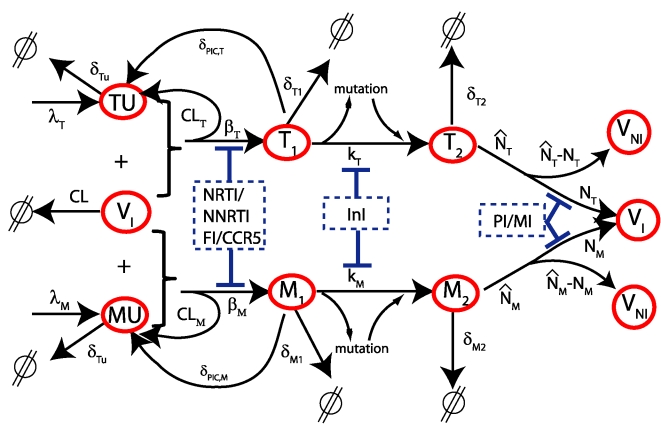
Simplified two stage virus dynamics model. Species (red cycles), reactions (black arrows), drugs and their interference in the life cycle of HIV (blue dashed box). Target cells (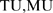) can become successfully infected by infective virus 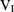 with lumped infection rate constants 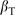 and 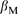, respectively, creating early infected cells 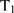 and 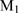. Infection can also be unsuccessful after the irreversible step of fusion (rate constant 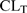 and 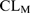), eliminating the virus and rendering the cell uninfected. Early infected cells 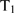 and 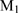 can destroy essential viral proteins or DNA prior to integration with rate constants 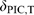 and 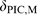 returning the cell to an uninfected stage. The genomic viral DNA can become integrated with rate constants 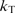 and 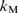 creating late infected cells 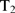 and 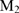, which can release new infectious- and non infectious virus 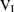 and 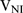 with rate constants 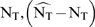 and 
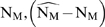
, respectively. Phenotypic mutation occurs at the stage of viral genomic integration 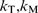 (see section ‘Development of a simplified two stage virus dynamics model’). All cellular compartments 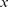 can get destroyed by the immune system with respective rate constants 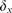 and the free virus gets cleared with rate constant 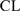.

In the following, we considered two types of target cells (T-Cells and a longer lived cell population, which we refer to as macrophages) and finally incorporated the viral mutation process (resulting from erroneous reverse transcription) into the overall model. Whether the longer lived cell population consists solely of macrophages *in vivo* remains unknown. There is, however, some evidence that the kinetic characteristics of the longer lived cell population are similar to those of the macrophage population [33]. The proposed simplified two-stage virus dynamics model is shown in Fig. 3. It comprises T-cells, macrophages, free non-infectious virus (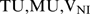, respectively), free infectious virus of mutant strain 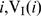, and four types of infected cells belonging to mutant strain 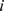: infected T-cells and macrophages *prior* to proviral genomic integration (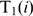 and 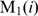, respectively) and infected T-cells and macrophages *after* proviral genomic integration (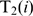 and 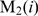, respectively). The rates of change of the different species in the reduced two-stage HIV model are given by the following system of ODEs:
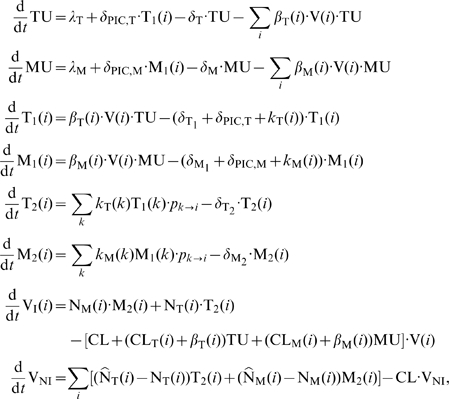
(6)where 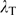 and 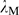 are the birth rates of uninfected T-cells and macrophages, and 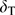 and 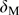 are their death rate constants. The parameters 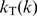 and 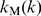 are the integration rate constants of mutant strain 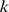. The parameters 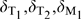 and 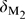 are the death rate constants of 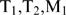 and 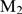 cells. The parameters 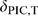 and 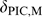 refer to the intracellular degradation of essential components of the pre-integration complex, e.g., by the host cell proteasome within early infected T-cells and macrophages respectively. 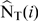 and 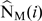 denote the total number of released infectious and non-infectious virus from late infected T-cells and macrophages of mutant strain 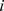 and 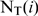 and 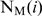 are the rates of release of infective virus (see Eq (5)). The parameters 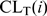 and 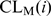 denote the clearance of mutant virus 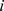 through unsuccessful infection of T-cells and macrophages respectively (see Eq. (4)) and the parameters 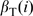 and 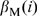 denote the successful infection rate constants of mutant virus 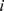 for T-cells and macrophages respectively. The parameter 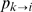 denotes the probability to mutate from strain 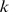 to strain 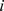 (to be defined below).

**Figure 4:**
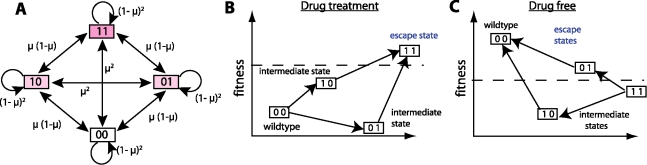
Fitness and possible mutational pathways. A: General transition pathways between wild type (00) and a fully drug resistant strain (11) that involves two partly-resistant intermediates (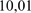). B: Fitness in the presence of a drug. C: Fitness in the absence of drugs. Dashed line: critical fitness that allows the strain to survive, i.e, 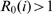.

The model enabled us to mechanistically incorporate the action of all drugs that are approved or in late clinical trial. The impact of a compound on a corresponding (lumped) parameter in the model is specified by 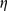:
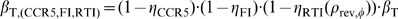
(7)

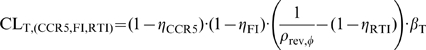
(8)

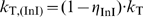
(9)

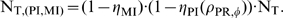
(10)The same quantities are defined for macrophages by replacing the subscript 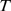 by 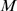; see Supplementary Text S1 for details. The overall viral dynamics model comprises a complete mutagenic graph. In HIV infection, genomic mutation occurs during the reverse transcription process [50]. The reverse transcriptase of HIV lacks a proof reading mechanism in contrast to host polymerase enzymatic reactions. However, viral proteins from newly mutated viral genomes are only produced after integration of the viral genome into the host cell DNA. The proteins required for the stable integration of the newly mutated viral genome originate from the founder virus. Therefore, phenotypically, drug resistance of new mutants will only be observed after integration, i.e., in the infectious stages 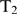 and 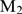. In total, the model includes 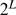 different viral strains 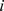 that contain point mutations in any pattern of the modelled 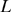 possible mutations. For two distinct mutations 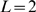, the mutagenic graph is shown in Fig. 4A. Each mutant 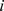 can mutate into every other mutant 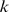 in one step. The probability 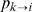 to mutate from a strain 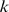 into another strain 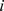 can be directly derived from the mutagenic pathways in Fig. 4A, i.e.,
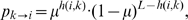
(11)where 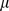 denotes the mutation probability per base and reverse transcription process (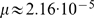 [50]), 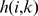 denotes the hamming distance between strain 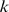 and strain 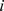, and 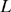 is the total number of different positions that are considered in our model. The phenotype of each mutant strain 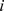 is modelled by introducing a selective disadvantage 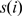, which denotes the loss of functionality (e.g., in the activity of some viral enzyme that is affected by the mutation) relative to the wild type, and a strain specific inhibitory activity (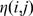) of treatment 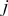 against the mutant strain 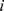. For example, the strain specific infection rate 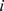 under a certain treatment 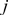 is given by 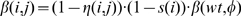, where 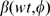 denotes the infection rate constant of the wild type 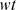 in the absence of drug 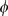 (given in Table 1). Since some viral strains are present only in very low copy numbers, we used a hybrid stochastic deterministic approach [51] to model the overall virus dynamics model (see *Materials and Methods* section for details).

**Table 1:** Model parameters generally used in simulations.

Parameter	Value	Reference	Parameter	Value	Reference
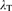	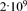	[57]	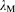	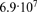	[33]
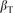	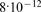	[32]	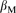	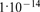	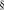
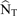	1000	[33]	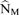	100	[33]
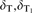	0.02	[33]	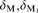	0.0069	[33]
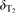	1	[73]	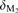	0.09	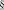
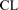	23	[73]	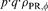	0.67	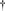
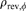	0.33	[74],[75]	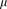	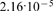	[50]
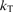	0.35	[75]	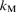	0.07	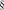
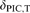	0.35	[75],[76]	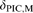	0.0035	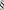
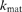	12	[34]	-	-	-

All parameters in units [1/day], except 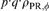 (unit less) and 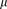 in 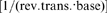. 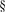 parameters chosen to reproduce clinical data. 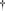 chosen according to the assumption that 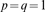 and utilizing parameters 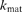 and 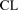 to determine 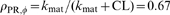.

### Reproductive capacity for predicting drug–specific impact on viral replication

The production of infectious offspring is crucial for the survival of a viral population. The phenotypic single-round infectivity assay measures the amount of infectious offspring after one round of replication. For a given drug, the assay quantifies the drug's efficacy by measuring the reduction in viral offspring relative to the drug-free situation. We defined a new quantity—termed the reproductive capacity 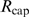
—, which transfers the principle of the phenotypic single-round infectivity assay into a mathematical term. Its definition involves the quasi-species distribution and the basic reproductive numbers of all pathogenic sub-stages. The reproductive capacity characterizes the fitness of a given state of the infection from the perspective of a potential treatment 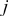 by quantifying the expected total number of offspring under the treatment 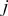.

The basic reproductive number 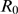 is a well characterized quantity in epidemiology that denotes the expected number of secondary infections caused by a single infected cell/virus [52]. If 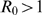 then the infection will spread, while for 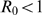 the infection will die out. The strain associated reproductive number 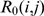 characterizes the fitness of a viral strain 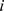 in a pharmacologically modified environment, specified by a drug treatment 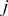. We used the ‘survival function’ approach [53] to calculate the reproductive numbers for mutant strains 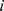 under treatment 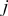. In our context, the survival function is of particular value, since it captures the possible event of mutation for all infective classes.

Based on the two-stage virus dynamics model, the basic reproductive number 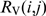 of a single virus of strain 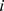 under treatment 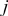 is given by
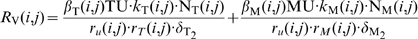
(12)with constants
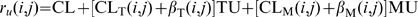
(13)

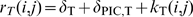
(14)

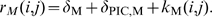
(15)Since infected cells are also pathogens, which can lead to a rebound of the disease even in the absence of any virus, we also determined their basic reproductive numbers under a given treatment 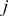. The basic reproductive numbers 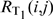 and 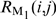 of the infectious stages 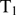 and 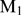, associated with the viral strain 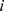, are given by
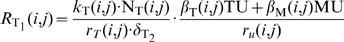
(16)

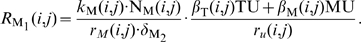
(17)Finally, the reproductive numbers 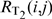 and 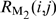 of the infectious stages 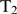 and 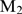, associated with the viral strain 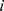, are given by
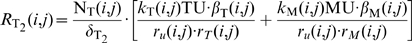
(18)

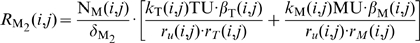
(19)We defined the reproductive capacity 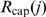 of the entire quasi-species ensemble under treatment 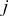 as the weighted sum of the basic reproductive numbers of all pathogenic stages of mutant strain 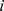, i.e., free virus, infected T-cells and infected macrophages, where the weights are the abundance of the corresponding pathogenic stage:
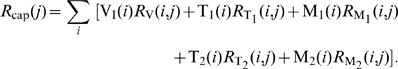
(20)The reproductive capacity 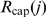 can be interpreted as the expected total number of infectious offspring that the infection produces in one round of replication under a certain treatment 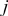, given the current state of the infection.

#### Relation to viral load and phenotypic/single-round infectivity assay

The viral load considers the total concentration of free virus, consisting of non-infectious virus 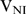 and infectious virus 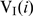, belonging to all mutant strains 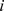. In contrast to the reproductive capacity, viral load does not assess the ability of distinct viral strains 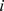 to replicate (in terms of 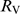). In mathematical terms, the viral load is given by
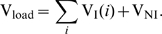
(21)The *in vitro* reproductive capacity, corresponding to the read-out of the phenotypic assay 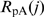 (under treatment 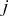) is conceptionally similar to Eq. (20). However, in comparison to the above defined *in vivo* measure, the *in vitro* measure would not take into account: (i) the clearance of any infective stage by the immune system (relating to the parameters 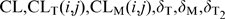, and 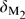), and (ii) the abundance of the different infected cell types (e.g., T-cells and macrophages). The assay measures one round of replication, denoted by 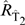, starting from a late stage infected cell 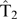. Mathematically expressed, the primary output is given by
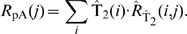
(22)


### Drug-class specific decay of viral load and reproductive capacity

**Figure 5:**
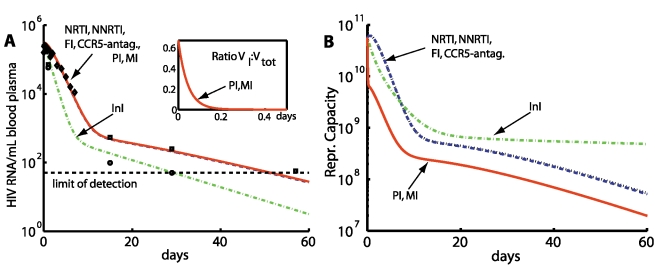
Decay of viral load and reproductive capacity after treatment initiation. A: Plasma virus load decay after treatment initiation. Integrase inhibitors (InI) produce a faster decay of virus load than all other compound classes. Red solid-, black dotted-, green dash-dotted- and blue dashed lines indicate simulation results with different inhibitor classes and parameters from Table 1. Black diamonds indicate median viral load data from [27] (PI monotherapy), numerically available in [70]. Black squares and black bullets indicate median viral load data from [21] (NRTI + background therapy and InI+background therapy, respectively). The horizontal dashed black line indicates the limit of detection of current assays (50 copies of HIV RNA per mL). Inset: Protease- and maturation inhibitors (PI and MI) change the ratio of infectious to total virus (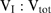). B: The evolution of the reproductive capacity (evaluated at the drug free state 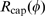) after treatment with different drug classes. Model parameters are as indicated in Table 1. The initial infection was assumed to consist of wild type only. Drug efficacy 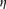 was assumed to be 100%. Total body virus has been converted to plasma viral load by assuming that the virus distributes into the plasma (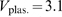 liters, which surrounds 2% of infected cells) and the interstitial space (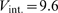 liters [71], which surrounds 98% of infected cells). The volume of distribution with reference to the plasma concentration has been calculated using the well-known formula vol. distr 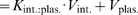, see e.g. [72], where 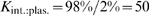. Finally, we assume that on average each virus contains 2 viral RNAs (which are measured [viral RNA/mL] plasma).

Application of drugs/drug classes changes the total size and the composition of the viral population. The impact of this change is typically evaluated in terms of the decay of the viral load over time. We used the reproductive capacity 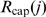 to also evaluate viral replication under various hypothetical treatments 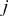. In Fig. 5, we predicted the impact of the different drug classes on the decay of the plasma viral load and the reproductive capacity 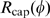, i.e., the fitness of the whole virus population, evaluated in the absence of drugs. As typically done, we assumed 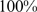 drug efficacy 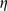.

In terms of the plasma viral load decay (Fig. 5A), we observe a faster initial decay for InIs in comparison to all other compound classes, in agreement with clinical data [21] and theoretical analysis [32],[33]. The onset of viral load decay is delayed for all other compound classes as observed clinically [12],[27], see also Figure S1. In agreement with clinical data [21], in the case of InI treatment, the second phase of viral decay starts earlier after treatment initiation and exhibits 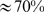 less viremia in comparison to other drug classes, but shows the same decay. Notably, the change of the ratio of infective virus-to-total virus (see Fig. 5, inset) upon PI or MI administration is not reflected by the total viral decay in the blood plasma.

Most noticeable, the reproductive capacity (Fig. 5B) discriminates between RTIs, FIs and CCR5-antagonists vs. InI vs. PIs and MIs. It can be seen, that protease and maturation inhibitors reduce 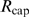 most efficiently initially and shift it to an overall lower level. Integrase inhibitors cause a slightly faster initial decay in 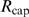, in comparison to RTIs, FIs and CCR5-antagonists, which consistent with the rapid decay in viral load (Fig. 5A). However, in contrast to viral load decay, the initial fast decay of 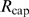 levels off and the second phase decay is flatter for InIs in comparison to RTIs, FIs, CCR5-antagonists, PIs and MIs. The effect of NRTIs, NNRTIs, CCR5 inhibitors and FIs on 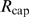 is comparable (Fig. 5B). Remarkably, these inhibitors induce an initial increase in 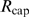 (see next section for details), followed by a slow first phase decay, followed by a second phase decay that is parallel to the decay of 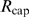 in the case of PI- and MI-treatment, sustaining overall higher levels of 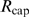 in comparison to PIs and MIs. In the next section, we further elucidate the reasons for these class-specific differences.

### Immune-system related clearance is critical determinant of drug-class specific decay

In view of the analysis performed in Fig. 5B, 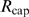 is directly correlated to the overall abundance of viral infectives (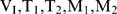).

**Figure 6:**
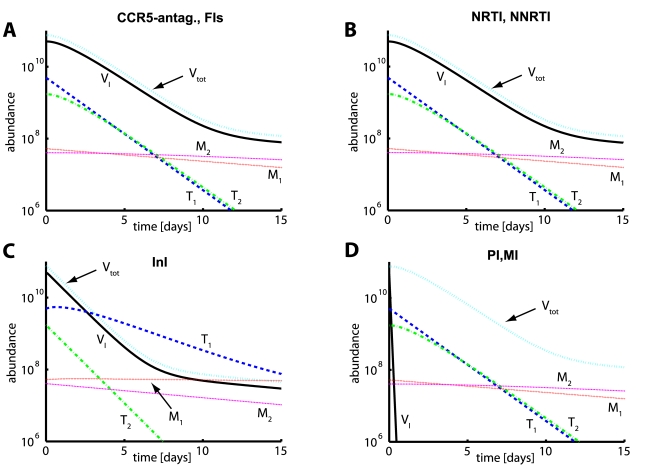
Decay of infective compartments after initiation of drug treatment. A: Decay of infective compartments after treatment with FI and CCR5-antagonists. B: Decay of infective compartments after treatment with NRTIs and NNRTIs. C: Decay of infective compartments after treatment with InIs. D: Decay of infective compartments after treatment with PIs.

PIs and MIs primarily act on infectious virus 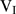 (see Fig. 5, inset), by reducing the proportionality factor (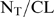 and 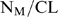) that determines the abundance of infectious virus in the first- and second phase decay (see Eq. (10)). The infectious virus 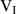 is rapidly cleared by the immune system [54]. Therefore, application of highly efficient PIs and MIs leads to a rapid reduction of infectious virus 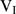, as illustrated in Fig. 6D and Fig. 5 (inset). This reduction is also reflected by the initial drop of 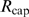 in Fig. 5B. In the case of PI and MI treatment, infected T-cells are quickly becoming the most abundant infectious compartment (Fig. 6D) and subsequently dominate the decay characteristics of 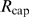 in Fig. 5B. In the final phase, late infected macrophages (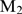) are becoming the most abundant compartment and thus dominate the decay of 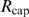 in the final phase.

Integrase inhibitors prevent the integration of the viral genome and thus prevent the transition of early infected cells (pre-integration, 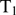 and 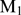) to late infected cells (post-integration, 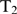 and 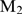), see Fig. 3. By inhibiting the transition from early to late infectious cells, integrase inhibitors increase the decay of late infected 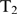-cells (see Fig. 6C). In the case of InI treatment, infectious virus 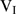 is initially proportional to 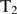, explaining the observed more rapid first-phase decline in 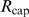 in Fig. 5B. However, blocking the transition from 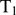 to 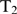 can also slow the decay of the 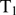-compartment, which might become more abundant than 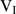 after the initial decay. In the final phase both 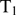 and 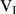 become proportional to- and remain more abundant than 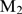, which explains the overall higher levels of 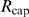 in the final phase (see Fig. 5B).

The effects of NRTIs, NNRTIs, CCR5 inhibitors and FIs on 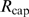 are comparable (Fig. 5B), as they primarily act on pre-integrative early infected cells (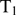 and 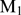). The difference between entry inhibitors and reverse transcriptase inhibitors is marginal, because the clearance of virus by infection is negligible compared to the clearance by the immune system (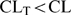 and 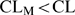). A positive result of entry inhibitors (FI/CCR5) and RTIs (NRTIs/NNRTIs) is an increased number of uninfected cells, which also results in an initial increase in the reproductive capacity 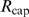 (see Fig. 5B). During treatment with NRTIs, NNRTIs, CCR5 inhibitors and FIs, infective virus 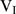 is the most abundant compartment. The decay in the first phase is proportional to the decay of the late infected cells, 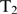. Once the abundance of 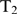 falls below 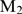, the decay of 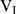 and thus 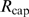 in Fig. 5B is proportional to the decay of late infected macrophages 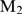.

### The pattern of virological removal influences the time to virological rebound after treatment application

In the following, we predict how the distinct viral dynamics after drug application affect drug efficacy *in vivo*. The long-term *in vivo* efficacy of an antiviral drug depends on many different factors, including the ability of the virus to adapt to the pharmacological challenge by developing resistance mutations. The ability to develop drug resistance is strongly dependent on the induced pattern of resistance mutations against a particular drug, but also on the velocity at which replication competent compartments (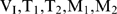) are removed from the body. Since anti-retroviral drug classes target different stages in the viral life cycle, they are likely to induce different patterns by which viral compartments are removed from the body (see Fig. 6) and might therefore exhibit different long-term *in vivo* efficacies.

To illustrate the sole impact of virological removal (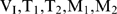) on resistance development and therefore on drug efficacy, we have intentionally chosen a simplistic, unified mutational landscape and considered the time to viral rebound as a long-term measure of efficacy. We denoted virological rebound, if the viral load reaches 90% of the pre-treatment viral load. We assumed that the drugs inhibited their targeted (lumped) parameter (see Eqs. (7)–(10)) by 90% in the wild type (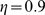), by 45% in a one-mutation strain (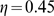) and are entirely inefficient in the double-mutant (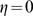). Drug-specific and more realistic mutational landscapes are possible, but in view of the current analysis (elucidating the impact of class-specific virological removal), they would blur the results.

In Table 2, the time to virological rebound for the different drug classes based on the above simplistic mutation model is reported. The virus generally rebounds to 90% of pre-treatment levels after 1–2 month of monotherapy, which is in the same order of magnitude as clinically observed rebound times [55]–[57]. Although inhibition 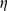 was assumed to be identical across all drug classes, the times to virological rebound differed. In particular, when resistance confers a marked loss in fitness (i.e. selective disadvantage = 30%), PIs show the longest time to virologically rebound, and the InIs the shortest.

**Table 2:** Virological rebound times resulting from distinct virological removal.

Drug/Selec. Disadvantage	30%	25%	20%	15%	10%	5%	1%
InI	48.13	44.44	41.33	38.70	36.43	34.65	33.25
FI/CCR5-antag.	53.71	47.81	43.09	39.57	36.47	33.77	32.06
NRTI/NNRTI	55.51	48.76	43.86	39.99	36.61	33.94	32.11
PI/MI	55.28	49.03	43.74	39.84	36.66	33.95	32.15

The time to virological rebound depends on both the cost of resistance (‘selective disadvantage’, 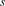) and the choice of drugs. Each table entry shows the time to virological rebound in [days] in an ensemble of 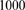 hybrid stochastic deterministic simulations, where we assumed that the efficacy of the drugs against the wild type was 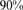. The drug was 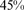 effective against an one-mutation strain and completely inefficient against the double-mutant. The fraction of non-infectious viruses (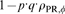) was set to one-third and the initial population was assumed to be all wild type. The viral load was said to be rebounded, if the viral load reached 90% of the pre-treatment viral load.

For integrase inhibitors, the difference between the decay of plasma viral load and their predicted long-term efficacy is quite pronounced. Their comparably shorter times to virological rebound are in strong contrast to their steep initial decrease of plasma viral load (see Fig. 5A), but consistent with the decay pattern of the reproductive capacity (Fig. 5B). For the EIs, RTIs, PIs and MIs, the predicted time to virological rebound is also much more consistent with the decay characteristics of the reproductive capacity (Fig. 5B) than with the decay pattern of total viral load (see Fig. 5A).

## Discussion

In clinical studies, the first approved integrase inhibitor, raltegravir, induced an extremely rapid decline in viral load when applied both as monotherapy [10] and in combination with an optimized NRTI background therapy [21]–[24]. While it was initially speculated that the observed decline might be a result of superior potency of raltegravir, it is now emerging that the viral decline in InI-based therapy could be a class-specific phenomenon [25],[26]. Moreover, superior potency of InIs (in terms of 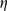) was not confirmed by single-round infectivity assays [14]. The mechanisms underlying the decay dynamics are still not clear [58] and controversially discussed [21],[32].

In [32], a two stage model of the viral replication cycle is presented, which explains the differences between the decay of viral load between RTIs and InIs based on the stage at which the drugs affect the dynamics of viral replication. The model explicitly distinguishes two viral stages, early-stage infected cells and late-stage infected target cells, which are specifically defined for a pair of drugs under examination. The authors further conclude that the viral dynamics produced by drugs from different anti-retroviral classes should not be directly compared to infer drug potency [33]. An alternative measure, as it is imperative for guiding drug discovery and prioritizing drug candidates in later development stages, is still lacking.

All currently approved antivirals exert their effect by inhibiting the replication of HIV. The extent at which replication is inhibited, is therefore a unifying indicator for drug efficacy across all drug classes. Replication assays, e.g., phenotypic assays [15] or replication capacity assays [59], analyze drug efficacy in terms of viral replication *in vitro*. The replicative fitness of HIV *in vivo*, however, depends on the interaction of a multitude of viral and host factors. Replication assays represent the dynamics of HIV under the assay conditions, which lack many host factors, in particular the immune responses to the infection. However, since it is particularly useful to compare compounds in terms of replication inhibition, we adopt the dynamic approach of replication assays to define the reproductive capacity 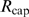. *In silico*, we are able to consider the host response to the viral infection and can thus extrapolate the replication approach from *in vitro* to *in vivo*. In [60], the authors used a similar approach to compare the effect of distinct antiviral classes utilizing age-structured models.

We derived a single detailed model of the viral replication cycle and deduced a reduced two stage model, which incorporates the action of *all* approved HIV drugs. Our two-stage model allows to predict the action of *any number* of drugs simultaneously, including common HAART cocktails, potentially belonging to different drug classes. In contrast, in [32], the stages of the two-stage model of viral replication are not specified *a priori* and have to be determined by the *two* drugs that are analyzed and compared.

Based on the proposed detailed and reduced model, we identify the following effects of currently approved drugs: EI and RTIs decrease the infection rate and thus the number of new infections. The impact on the release of new virus (and virus decline) is therefore delayed by the viral life cycle. MIs and PIs do not interfere with the total amount of virus that is being released, but rather shift the ratio of infective to total virus, 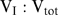 (see Fig. 5, inset), which is not directly reflected by total plasma viral load. Since the kinetics of the free virus are rapid [54], this has an immediate impact on the number of new infections. Subsequently, this impact on the number of new infections affects the total viral release (and thus total plasma virus load) in a similar manner as EIs and RTIs, creating a ‘shoulder’ phase. Hence, we obtain
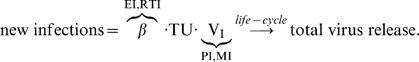
(23)In our model, EIs, RTIs, PIs and MIs produce an identical decay of plasma viral load (see Fig. 5A), when assuming 100% inhibition, respectively. In particular, the onset of viral load decay is similarly delayed (‘shoulder phase’) with these inhibitors (see Figure S1), in agreement with clinical observations [12],[27]. Previously discussed theoretical differences in the viral response between RTIs and PIs (see Eq. (5.7) vs. Eq. (5.16) in [61]) yield similar dynamics when more recent (higher) estimates of viral clearance are used [54].

In contrast to other inhibitor classes, InIs decrease the amount of late infected cells (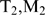) (see Fig. 2), which has an immediate impact on total virus release, i.e.,
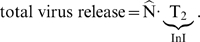
(24)The impact of InIs on viral load decay is immediate and not delayed by the viral replication cycle as in the case of all other compounds [12],[27]. Thus, the onset of observed total viral decay is faster for InIs than for other compounds, irrespective of their potency (which was set equal for all compounds in Fig. 5A). Furthermore, the decay of viral load in the first phase is steeper for InIs in comparison to other inhibitor classes (see Fig. 5A). The viral load decline in the first phase is proportional to the decay of the late infected T-cells 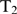 (see Fig. 6). Sedaghat et al. [32] derived analytical solutions for the viral decay dynamics after InI and RTI treatment (see Eqs. (9) and (10) in [32]), which demonstrate that the viral decay after InI treatment is determined by the death rate of late infected cells (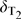), while in the case of RTI treatment, the decay is determined by the “flushing-out” of the early infected cells (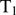) and the death rate of the late infected cells 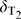, leading to overall faster viral declines in the case of InI treatment in the first phase.

The long-term *in vivo* efficacy of an antiviral drug depends on many different factors, particularly the ability of the virus to adapt to the pharmacological challenge by developing resistance mutations. The ability to develop drug resistance is strongly dependent on the induced pattern of resistance mutations against a particular drug, but might also be influenced by the velocity at which replication competent compartments are removed from the body. However, viral load decay focusses on only one single variable, namely the total output of virus, whereas other infectious stages (e.g. 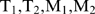) remain ‘hidden’. Furthermore, the ratio of infective virus-to-total virus (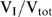) is not resolved, which might underestimate the long-term efficacy of PIs and MIs that target this ratio (see Table 2 in relation to Fig. 5A). In the section ‘The pattern of virological removal influences the time to virological rebound after treatment application’, we have compared the impact of drug-class specific removal patterns on the long-term efficacy of antivirals (in terms of resistance development). We showed that although inhibition 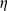 was assumed to be identical across all drug classes, the times to virological rebound (used as a measure of long-term efficacy) differed, with PIs showing the longest time to virologically rebound, and InIs the shortest.

The reproductive capacity has been monitored over time in Fig. 5B to depict class-specific long-term efficacy of antivirals based on the hosts' ability to clear the targeted infectant in the viral life cycle. The main conclusion is that the long-term efficacy is larger for compounds that target viral life-stages that are cleared at a fast rate. It is generally assumed that the free virus is cleared at the fastest rate [27],[54]. Since MIs and PIs reduce the production of infective virus 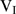 (see Fig. 2), they reduce the virus' ability to produce offspring faster than all other drug classes. Furthermore, since resistance development is correlated with the extent of replication, we infer that PIs and MIs, based on their viral target, are the most efficient drug classes in terms of reducing the probability of resistance development. This assumption correlates well with the observed rebound times in Table 2 and is also supported by the fact that the introduction of PIs marked the success of HAART [1].

During drug discovery, the pre-clinical- and the clinical development process, *in vitro* surrogate measures or *in vivo* drug efficacy measures are important to prioritize drug candidates.

The mechanistic mode of action of a compound at its target site can be elucidated by cell free assays that use purified viral protein, e.g. reverse transcriptase for RTIs. The influence of viral mutation, the immune system and pharmacokinetics are absent in this type of assay. However, it is possible to deduce the pharmacodynamic mode (e.g. Eq. (1), see also [41]) and thus the parameter 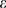 from these types of assays, which denotes the extent of inhibition of the molecular process by the compound. Mathematical models of HIV dynamics use a minimal number of parameters, making them suitable for parameter fitting and comparison with clinical data. The parameters used in the models are often lumped, summarizing many viral processes. For example, binding, fusion and reverse transcription are part of the infection rate 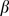 (see Eq. (3)). Inhibition of lumped model parameters (denoted by 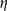) might therefore differ from inhibition of the molecular process 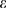, which is measured by cell-free *in vitro* assays. We have provided equations (Eqs. (S24) and (S31), Supplementary Text S1) that enable the use of pharmacodynamic information 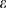, derived from cell free assays (inhibition of the targeted molecular process), in a (lumped) mathematical model of HIV dynamics (utilizing 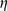).

The presented model can be extended to incorporate drug-specific escape pathways [62],[63] or realistic time-varying drug pharmacokinetics [41]. If *in vivo* pharmacokinetic data is available (in terms of time-varying concentrations 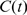 in Eq. (1)), then extrapolation from *in vitro* to *in vivo* is possible and the mechanistic understanding of drug effects, its parametrization and extrapolation is facilitated. For RTIs and PIs, we found a nonlinear relationship between 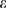 and 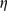 (see Eqs. (S24) and (S31), Supplementary Text S1). Utilization of Eqs. (S24) and (S31) allows to simulate drug effects based on their mechanistic understanding in a lumped model, that can be compared with clinical data.

The model can also be extended to include latently infected cells (very long lived infected cells). We did not consider them in this study, since they are expected to contribute little to the dynamics analyzed herein (the first and the second decay phase).

The reproductive capacity is a useful concept to analyze and monitor drug efficacy *in silico*. In its current form, the reproductive capacity requires detailed knowledge about (i) the composition of the viral population, and (ii) the fitness of the different viral strains under a given treatment (reproductive numbers, Eqs. (12) and (16)–(19)).

The fitness of certain viral strains can be assessed *in vitro*, e.g., by phenotypic assays. We model strain specific fitness 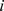 under treatment 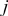, in terms of two parameters: the selective disadvantage 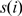, which denotes the loss in replication of mutant 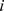, relative to the wild type; and the efficacy of treatment 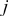 against mutant 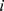 in terms of the parameter 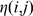. The selective disadvantage can, e.g., be estimated by performing a phenotypic assay with a certain mutant virus 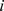 in the absence of drug and then comparing it to the assay with the wild type. The parameter 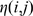 is already being assessed in practice (e.g., [15]), usually in terms of a fold increase in 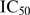.

Acquisition of detailed knowledge about the composition of the viral population might, due to recent advances in sequencing technology [64]–[67], become feasible in the future. However, novel sequencing technology requires large amounts of viral RNA, which cannot be derived when the viral load is below the limits of detection.

## Materials and Methods

### Realization of hybrid simulations

The overall virus dynamics in our model comprise different viral strains with copy numbers that can vary over several orders of magnitude. For this reason we have chosen a hybrid (stochastic deterministic) setting for numerical simulation. This approach (i) takes stochastic fluctuations in the slow reaction processes into account; and (ii) reduces the computational costs for the simulation of the fast (deterministic) system dynamics. We used the direct hybrid method proposed in [51]. Elementary reactions were treated stochastically whenever their propensity function or the quantity of at least one of their reactants was below a certain threshold (for all numerical simulations this threshold was set to 5). For the numerical integration of the deterministic part of the system, we implemented a solver in C++ that is based on numerical differentiation formulas [68] and uses strategies for error control and step size control comparable to ode15s in Matlab [69]. To generate the data for Table 2, we performed 1000 hybrid simulations for each condition. With realization start (*t* = 0), the effects of the drug treatment were simulated until the viral population size reached 90% of its pre-treatment value, i.e., virological rebound occurred. During a simulation, the stochastic partitioning of the reaction system was dynamically updated and stochastic reaction events were realized accordingly. Every numerical calculation was computed with a relative error tolerance of 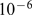 and an absolute error tolerance of 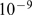. The hybrid simulations for Table 2 were performed on two Intel Quad-Core Xeon E5345 processors with 2.33 GHz and 32 GB RAM, which took nearly 46 hours in total or approximately 6 seconds per simulation, respectively.

## Supporting Information

Text S1 This file contains the derivation of the simplified model (Fig. 3) from the detailed model (Fig. 1).
(0.30 MB PDF)

Figure S1 Delay in the onset of viral load decay, exemplified for PI treatment. Simulation results (red line) using the novel two stage virus dynamics model and simulating 100% effective PI treatment are shown together with median clinical data (black diamonds) from PI (RTV) monotherapy.
(0.91 MB EPS)
