# Supplementary material for: Drug-Class Specific Impact of Antivirals on the Reproductive Capacity of HIV
Source: PLoS Comput Biol. 2010 Mar 26;6(3):e1000720. doi: 10.1371/journal.pcbi.1000720 (PMC2845651; doi:10.1371/journal.pcbi.1000720)
Supplement: Text S1 — This file contains the derivation of the simplified model (Fig. 3) from the detailed model (Fig. 1). (0.30 MB PDF) [file pcbi.1000720.s001.pdf]

Supplementary Material:
Drug-Class Specific Impact of Antivirals on the Reproductive Capacity of HIV

Max von Kleist Stephan Menz Wilhelm Huisinga

S1 Supplementary Text

Detailed Model

The rate of change in the detailed model of the viral life cycle is specified by the following system of ordinary differential equations (ODEs):

$$\begin{aligned}
\frac{\mathrm{d}}{\mathrm{d}t}\mathrm{TU} &= \lambda_\mathrm{T} - k_\mathrm{on}\mathrm{TU}\cdot\mathrm{V_I} + k_\mathrm{off}[\mathrm{V_I}:\mathrm{TU}] \\
&\quad +\delta_\mathrm{RNA}\cdot\mathrm{T_{RNA}} + \delta_\mathrm{PIC,T}\cdot\mathrm{T_1} - \delta_\mathrm{T}\cdot\mathrm{TU} && \text{(S1)}\\
\frac{\mathrm{d}}{\mathrm{d}t}[\mathrm{V_I}:\mathrm{TU}] &= k_\mathrm{on}\mathrm{TU}\cdot\mathrm{V_I} - k_\mathrm{off}[\mathrm{V_I}:\mathrm{TU}] - k_\mathrm{fus}[\mathrm{V_I}:\mathrm{TU}] && \text{(S2)}\\
\frac{\mathrm{d}}{\mathrm{d}t}\mathrm{T_{RNA}} &= k_\mathrm{fus}\cdot[\mathrm{V_I}:\mathrm{TU}] - (\delta_\mathrm{T} + \delta_\mathrm{RNA} + k_\mathrm{rev})\mathrm{T_{RNA}} && \text{(S3)}\\
\frac{\mathrm{d}}{\mathrm{d}t}\mathrm{T_1} &= k_\mathrm{rev}\cdot\mathrm{T_{RNA}} - (\delta_\mathrm{T} + \delta_\mathrm{PIC,T} + k_\mathrm{T})\mathrm{T_1} && \text{(S4)}\\
\frac{\mathrm{d}}{\mathrm{d}t}\mathrm{T_2} &= k_\mathrm{T}\cdot\mathrm{T_1} - \delta_{\mathrm{T_2}}\cdot\mathrm{T_2} && \text{(S5)}\\
\frac{\mathrm{d}}{\mathrm{d}t}\mathrm{V_{IM}} &= p\cdot\widehat{\mathrm{N_T}}\cdot\mathrm{T_2} - (\mathrm{CL} + k_\mathrm{mat})\mathrm{V_{IM}} && \text{(S6)}\\
\frac{\mathrm{d}}{\mathrm{d}t}\mathrm{V_D} &= (1-p)\cdot\widehat{\mathrm{N_T}}\cdot\mathrm{T_2} + (1-q)\cdot k_\mathrm{mat}\cdot\mathrm{V_{IM}} - \mathrm{CL}\cdot\mathrm{V_D} && \text{(S7)}\\
\frac{\mathrm{d}}{\mathrm{d}t}\mathrm{V_I} &= q\cdot k_\mathrm{mat}\cdot\mathrm{V_{IM}} - \mathrm{CL}\cdot\mathrm{V_I} - k_\mathrm{on}\mathrm{TU}\cdot\mathrm{V_I} + k_\mathrm{off}[\mathrm{V_I}:\mathrm{TU}]. && \text{(S8)}
\end{aligned}$$

The definitions of the parameters and variables are given in the *Results* section of the main article.

Mechanistic Parameter Lumping

Under the reasonable assumption that $k_\mathrm{off} \gg k_\mathrm{fus}$, we get

$$\frac{[\mathrm{V_I}:\mathrm{TU}]}{\mathrm{TU}\cdot\mathrm{V_I}} = \frac{1}{\mathrm{K_D}} \qquad \text{(S9)}$$

from Eq. (S2), where $\mathrm{K_D} = \frac{k_\mathrm{off}}{k_\mathrm{on}}$. Applying the quasi-steady state assumption to $\mathrm{T_{RNA}}$ yields

$$\frac{\mathrm{T_{RNA}}}{[\mathrm{V_I}:\mathrm{TU}]} = \frac{k_\mathrm{fus}}{k_\mathrm{rev} + \delta_\mathrm{RNA}}, \qquad \text{(S10)}$$

where we exploited that $\delta_{\mathrm{RNA}} \gg \delta_{\mathrm{T}}$. We can now determine the effective infection rate by parameterizing the rate of new infections $k_{\mathrm{rev}} \cdot \mathrm{T_{RNA}}$ in terms of $\mathrm{TU} \cdot \mathrm{V_I}$. Using the above relationsships we obtain

$$k_{\mathrm{rev}} T_{\mathrm{RNA}} = k_{\mathrm{rev}} \frac{\mathrm{T_{RNA}}}{[\mathrm{V_I} : \mathrm{TU}]} \cdot \frac{[\mathrm{V_I} : \mathrm{TU}]}{\mathrm{TU} \cdot \mathrm{V_I}} \cdot \mathrm{TU} \cdot \mathrm{V_I} \tag{S11}$$

$$= \underbrace{\frac{k_{\mathrm{fus}}}{\mathrm{K_D}} \cdot \frac{k_{\mathrm{rev}}}{k_{\mathrm{rev}} + \delta_{\mathrm{RNA}}}}_{\beta} \cdot \mathrm{TU} \cdot \mathrm{V_I}. \tag{S12}$$

The parameter β describes the overall (lumped) basic infection rate up to the early infectious stage $\mathrm{T_1}$. Since we want to eliminate the two intermediate stages $[\mathrm{V_I} : \mathrm{TU}]$ and $\mathrm{T_{RNA}}$, we also have to take into account the clearance of viral particles associated with these stages. Aiming at a lumped clearance in terms of $\mathrm{V_I} \cdot \mathrm{TU}$ we exploit Eqs. (S9), (S10) and (S12) to obtain

$$\begin{aligned} \mathrm{CL_T} \cdot \mathrm{V_I} \cdot \mathrm{TU} &= (\delta_{\mathrm{T}} + \delta_{\mathrm{RNA}}) \mathrm{T_{RNA}} \\ &= \left(\frac{k_{\mathrm{fus}}}{\mathrm{K_D}} \cdot \frac{\delta_{\mathrm{RNA}}}{k_{\mathrm{rev}} + \delta_{\mathrm{RNA}}} \right) \cdot \mathrm{V_I} \cdot \mathrm{TU} \\ &= \frac{\delta_{\mathrm{RNA}}}{k_{\mathrm{rev}}} \cdot \beta \cdot \mathrm{V_I} \cdot \mathrm{TU}, \end{aligned} \tag{S13}$$

where we have assumed that $[\mathrm{V_I} : \mathrm{TU}] \ll \mathrm{V_I}$ and $\delta_{\mathrm{T}} \ll \delta_{\mathrm{RNA}}$. In most models, this term is not explicitly considered, but rather modelled as part of the overall constant viral clearance CL, thus ignoring the possible impact of unsuccessful infection on viral clearance.

Let us define the probability $\rho_{\mathrm{rev},\phi}$ that reverse transcription is successfully finished in the absence of drugs:

$$\rho_{\mathrm{rev},\phi} = \frac{k_{\mathrm{rev}}}{k_{\mathrm{rev}} + \delta_{\mathrm{RNA}}}, \tag{S14}$$

where we again used $\delta_{\mathrm{T}} \ll \delta_{\mathrm{RNA}}$. Then Eq. (S13) becomes

$$\mathrm{CL_T} = \left(\frac{\beta}{\rho_{\mathrm{rev},\phi}} - \beta \right). \tag{S15}$$

Along the same lines, we eliminate $\mathrm{V_{IM}}$ by assuming quasi-steady state conditions for the viral stage in Eq. (S6)

$$\mathrm{V_{IM}} = p \cdot \frac{\widehat{\mathrm{N_T}}}{\mathrm{CL} + k_{\mathrm{mat}}} \mathrm{T_2}. \tag{S16}$$

Then the amount of maturated, released virions $q \cdot k_{\mathrm{mat}} \cdot \mathrm{V_{IM}}$ in terms of $\mathrm{T_2}$ is given by

$$q \cdot k_{\mathrm{mat}} \mathrm{V_{IM}} = \underbrace{q \cdot p \cdot \frac{k_{\mathrm{mat}}}{\mathrm{CL} + k_{\mathrm{mat}}} \cdot \widehat{\mathrm{N_T}}}_{\mathrm{N_T}} \cdot \mathrm{T_2}. \tag{S17}$$

This term can now be used to replace $\mathrm{V_{IM}}$ in Eqs. (S6)–(S8). Finally we subsume the non-infectious virus $\mathrm{V_{NI}} = \mathrm{V_{IM}} + \mathrm{V_D}$.

Lumped Model

Applying the lumping process, the simplified model is defined by the following system of ODEs:

$$\begin{aligned}
\frac{\mathrm{d}}{\mathrm{d}t}\mathrm{TU} &= \lambda_{\mathrm{T}} + \delta_{\mathrm{PIC,T}} \cdot \mathrm{T}_1 - \delta_{\mathrm{T}} \cdot \mathrm{TU} - \beta \cdot \mathrm{V}_{\mathrm{I}} \cdot \mathrm{TU} \\
\frac{\mathrm{d}}{\mathrm{d}t}\mathrm{T}_1 &= \beta \cdot \mathrm{V}_{\mathrm{I}} \cdot \mathrm{TU} - (\delta_{\mathrm{T}_1} + \delta_{\mathrm{PIC,T}} + k_{\mathrm{T}})\,\mathrm{T}_1 \\
\frac{\mathrm{d}}{\mathrm{d}t}\mathrm{T}_2 &= k_{\mathrm{T}}\mathrm{T}_1 - \delta_{\mathrm{T}_2}\mathrm{T}_2 \qquad \text{(S18)} \\
\frac{\mathrm{d}}{\mathrm{d}t}\mathrm{V}_{\mathrm{I}} &= \mathrm{N}_{\mathrm{T}} \cdot \mathrm{T}_2 - \big(\mathrm{CL} + (\mathrm{CL}_{\mathrm{T}} + \beta)\mathrm{TU}\big) \cdot \mathrm{V}_{\mathrm{I}} \\
\frac{\mathrm{d}}{\mathrm{d}t}\mathrm{V}_{\mathrm{NI}} &= \big(\widehat{\mathrm{N}_{\mathrm{T}}} - \mathrm{N}_{\mathrm{T}}\big)\mathrm{T}_2 - \mathrm{CL} \cdot \mathrm{V}_{\mathrm{NI}} \qquad \text{(S19)}
\end{aligned}$$

This model can now be extended for, e.g., macrophages or mutational dynamics to derive the model in Fig. 3.

Parameters

Experimental data and mathematical analysis has suggested that the lifetime of virus producing cells in the first phase of viral decay (dictated by δ_{T_2}) is remarkably short (see e.g. [1–3]). The decay rate of the virus producing T-cell compartment has been estimated to be ≈1/day [4]. On the other hand, the lifespan of healthy T-cells has been reported to be many magnitudes larger [5]. Hence, $\delta_{\mathrm{T}_2} \gg \delta_{\mathrm{T}}$. T_2 cells (infected T-cells with integrated viral genome) are assumed to express far more HIV proteins than T_1 cells (infected T-cells with unintegrated viral genome), as virus proteins are mainly generated from the integrated viral genomic template and then expressed at the cell surface. Therefore, late infected cells (T_2) are far more prone to immune detection and clearance (which is denoted by δ_{T_2}). Therefore, we chose $\delta_{\mathrm{T}_2} \gg \delta_{\mathrm{T}_1}$. This assumption is also supported by the parameter choices made in related publications [6, 7].

The time scale of RNA degradation is seconds to a couple of hours (e.g. [8, 9]), while healthy cells are believed to be degraded within several days (e.g. [5]). Hence, we chose $\delta_{\mathrm{RNA}} \gg \delta_{\mathrm{T}}$.

The novel parameter k_{T} can be derived by combining information from the literature [10, 11] with our model. Zhou et al. [11] have measured the decay of the pre-integration complex and estimated a halflife of 2 days ($\delta_{\mathrm{PIC,T}} = 0.35$ [1/day]). This value was later confirmed by [10]. Furthermore, Zhou et al. [11] stated that approximately 50% of viral PICs undergo degradation in infected cells after infection. Therefore, the fraction of successful integration $\rho_{\mathrm{integr.}}$ is also approximately 50%. From our model, we derive

$$\frac{k_{\mathrm{T}}}{k_{\mathrm{T}} + \delta_{\mathrm{PIC,T}} + \delta_{\mathrm{T}}} = \rho_{\mathrm{integr.}} \approx 0.5. \qquad \text{(S20)}$$

Assuming $\delta_{\mathrm{PIC,T}} \gg \delta_{\mathrm{T}}$, we finally obtain $k_{\mathrm{T}} = 0.35$ [1/day]. Values for $\rho_{\mathrm{rev},\phi}$ range from 15% [12] to 50% [11]. The parameter k_{mat} has been reported to be in the range of 12 [1/day] [13]. If we initially set $q = p = 1$, and assume that infected cells that do not express viral proteins are cleared at the same rate as uninfected cells ($\delta_{\mathrm{T}_1} = \delta_{\mathrm{T}}$) then the remaining number of unknown parameters ($\beta, \widehat{\mathrm{N}}, \lambda_{\mathrm{T}}, \delta_{\mathrm{T}}, \delta_{\mathrm{T}_2}, \mathrm{CL}$) is the same as in the standard models [3].

Effect of Compounds on Lumped Parameters

In the following we will denote the local effects of compounds on the targeted processes in the detailed model by ε, while effects of drugs on lumped parameters in the simplified model (Fig. 3) will be denoted by η.

Entry inhibitors and RTIs. We have determined the basic infection rate constant β in Eq. (S12). Locally, CCR5-inhibitors, FI and RTIs inhibit binding k_{on}, fusion k_{fus} and reverse transcription k_{rev}. The rate of successful infection $\beta_{\text{CCR5,FI,RTI}}$ in the presence of inhibitors is given by

$$\beta_{\text{CCR5,FI,RTI}} = (1-\varepsilon_{\text{FI}}) \cdot (1-\varepsilon_{\text{CCR5}}) \cdot \frac{k_{\text{fus}}}{\text{K}_\text{D}} \cdot \frac{(1-\varepsilon_{\text{RTI}}) \cdot k_{\text{rev}}}{(1-\varepsilon_{\text{RTI}}) \cdot k_{\text{rev}} + \delta_{\text{RNA}}}, \tag{S21}$$

where $(1-\varepsilon) \in [0,1]$ denotes the residual activity of the targeted processes in the presence of the inhibitor. From the above equation it becomes clear that the local effect on the detailed parameters and the global effect on the lumped parameters are identical for FIs and CCR5-antagonists, i.e., $\varepsilon_{\text{FI}} = \eta_{\text{FI}}$ and $\varepsilon_{\text{CCR5}} = \eta_{\text{CCR5}}$. For RTIs, however, the overall effect on the rate of successful infection β depends on the decay rate constant δ_{RNA} of viral RNA.

Let $\rho_{\text{rev,RTI}}$ denote the probability that reverse transcription is successfully completed in the presence of RTIs:

$$\rho_{\text{rev,RTI}} = \frac{(1-\varepsilon_{\text{RTI}}) \cdot k_{\text{rev}}}{(1-\varepsilon_{\text{RTI}}) \cdot k_{\text{rev}} + \delta_{\text{RNA}}}. \tag{S22}$$

The effect of RTIs on the lumped infection rate β can now be interpreted as the reduction in the probability to successfully complete reverse transcription

$$1 - \eta_{\text{RTI}}(\rho_{\text{rev},\phi}) = \frac{\rho_{\text{rev,RTI}}}{\rho_{\text{rev},\phi}}, \tag{S23}$$

resulting from the reduced rate of reverse transcription, which increases the likelihood that parts of the viral RNA are degraded before being transformed into DNA:

$$(1-\eta_{\text{RTI}}(\rho_{\text{rev},\phi})) = \frac{(1-\varepsilon_{\text{RTI}}) \cdot k_{\text{rev}}}{(1-\varepsilon_{\text{RTI}}) \cdot k_{\text{rev}} + \delta_{\text{RNA}}} \cdot \frac{k_{\text{rev}} + \delta_{\text{RNA}}}{k_{\text{rev}}} = \frac{1}{\rho_{\text{rev},\phi} + \frac{1-\rho_{\text{rev},\phi}}{(1-\varepsilon_{\text{RTI}})}}. \tag{S24}$$

Therefore, for RTIs, we derive a non-linear relationship between inhibition of reverse transcription and inhibition of infection, in contrast to CCR5-antagonists and FIs. The relation between the local effect of RTIs on reverse transcription ε_{RTI} and the effect of RTIs on the infection rate $\eta_{\text{RTI}}(\rho_{\text{rev},\phi})$ is illustrated in Fig. S1A. It can be seen that the non-linearity between $(1-\eta_{\text{RTI}})$ and $(1-\varepsilon_{\text{RTI}})$ is most pronounced if reverse transcription has a high likelihood of being finalized in the absence of drug, i.e, $\rho_{\text{rev},\phi} \approx 1$. In this case $(1-\eta_{\text{RTI}}) > (1-\varepsilon_{\text{RTI}})$ or $\varepsilon_{\text{RTI}} > \eta_{\text{RTI}}$, implying that the effect of RTIs on the infection rate is smaller than the effect on reverse transcription. If reverse transcription is rarely finalized in the absence of drug, i.e., $\rho_{\text{rev},\phi} \approx 0$, then the effects on reverse transcription and on the infection rate become identical, $(1-\eta_{\text{RTI}}) = (1-\varepsilon_{\text{RTI}})$ or $\varepsilon_{\text{RTI}} \approx \eta_{\text{RTI}}$.

Using Eq. (S24), we obtain the rate of (successful) infection $\beta_{\text{CCR5,FI,RTI}}$ in the presence of CCR5-antagonists, FIs and RTIs:

$$\beta_{\text{CCR5,FI,RTI}} = (1-\eta_{\text{CCR5}}) \cdot (1-\eta_{\text{FI}}) \cdot (1-\eta_{\text{RTI}}(\rho_{\text{rev},\phi})) \cdot \beta, \tag{S25}$$

where β denotes the basic infection rate in the absence of inhibitors. The equation above implies that the effect of CCR5-antagonists, FIs and RTIs can be modeled at the level of infection. We also illustrated that in the case of RTIs, there might be a discrepancy between the local effects on the enzyme (reverse transcriptase) and the effects on the lumped infection rate β. To elucidate whether this discrepancy has any consequence *in vivo*, it is necessary to determine which fraction $\rho_{\text{rev},\phi}$ of the viral RNA that enters the cell is usually transcribed into full length DNA.

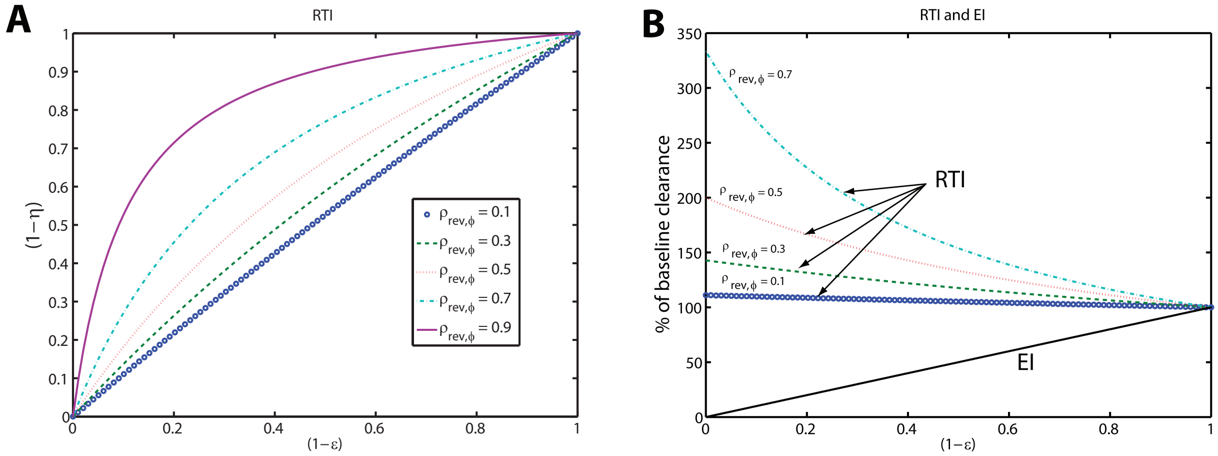

Figure S1. The effect of RTIs depends on $\rho_{rev,\phi}$. A: Correlation between effect of RTIs on the reverse transcription $(1-\varepsilon_{\mathrm{RTI}})$ and effect on the infection rate $(1-\eta_{\mathrm{RTI}})$ for a parameter range of $\rho_{\mathrm{rev},\phi}$ (the probability to finalize reverse transcription in the absence of RTIs). B: Effect of RTIs and EIs on the clearance through unsuccessful infection $\mathrm{CL_T}$.

Using the same considerations as above, we can derive the effect of CCR5 inhibitors, FI and RTIs on the clearance through unsuccessful infection (cf. Eq. (S13)):

$$\begin{aligned}\mathrm{CL_{T,CCR5,FI,RTI}} &= (1-\varepsilon_{\mathrm{CCR5}})\cdot(1-\varepsilon_{\mathrm{FI}})\cdot\left(\frac{k_{\mathrm{fus}}\cdot\rho_{\mathrm{rev,RTI}}}{\mathrm{K_D}\cdot\rho_{\mathrm{rev,RTI}}}-\frac{k_{\mathrm{fus}}}{\mathrm{K_D}}\cdot\rho_{\mathrm{rev,RTI}}\right)\\ &= (1-\eta_{\mathrm{CCR5}})\cdot(1-\eta_{\mathrm{FI}})\cdot\left(\frac{\beta}{\rho_{\mathrm{rev},\phi}}-(1-\eta_{\mathrm{RTI}})\cdot\beta\right). \qquad \text{(S26)}\end{aligned}$$

From this equation it is clear that effective RTIs ($\eta_{\mathrm{RTI}}\approx 1$) significantly contribute to the clearance of virus through unsuccessful infection, whereas FIs and CCR5-antagonists lower the clearance of virus through unsuccessful infection, because they inhibit viral infection at steps that precede the step of viral RNA destruction δ_{RNA} (see Fig. S1B).

Most models (see e.g. [14]) do not consider the clearance of virus through (unsuccessful) infection $\mathrm{CL_T}$ explicitly and treat it as part of the constant virus clearance CL. However, as we demonstrated, inhibitors can have an effect on the clearance. If one is only interested in the additional clearance, and not the baseline clearance of virus through unsuccessful infection, it can be derived by subtracting $\mathrm{CL_{T,CCR5,FI,RTI}}-\mathrm{CL}_{\mathrm{T},\phi}$, using Eqs. (S15) and (S26).

Integrase inhibitors. InIs inhibit the integration of viral DNA into the host DNA. We did not perform parameter lumping at this stage and therefore $\eta_{\mathrm{InI}}=\varepsilon_{\mathrm{InI}}$. The rate of integration is altered in the presence of InIs, according to

$$k_{\mathrm{T,InI}}=(1-\eta_{\mathrm{InI}})\cdot k_{\mathrm{T}}. \qquad \text{(S27)}$$

Protease and maturation inhibitors. PIs inhibit the protease of HIV, resulting in a decreased maturation rate constant k_{mat}, and MIs decrease the probability that virions maturate normally q. Recalling Eq. (S17), the total input into the compartment of infective virus $\mathrm{V_I}$ is described by

$$q\cdot k_{\mathrm{mat}}\mathrm{V_{IM}} = (1-\varepsilon_{\mathrm{MI}})\cdot q\cdot p\cdot\frac{(1-\varepsilon_{\mathrm{PI}})\cdot k_{\mathrm{mat}}}{\mathrm{CL}+(1-\varepsilon_{\mathrm{PI}})\cdot k_{\mathrm{mat}}}\cdot\widehat{\mathrm{N}}_{\mathrm{T}}\cdot\mathrm{T_2}. \qquad \text{(S28)}$$

In the case of MIs we have $\varepsilon_{\mathrm{MI}} = \eta_{\mathrm{MI}}$. However, in the case of PIs, their effect depends on the clearance of free virus CL. Similarly to the case with RTIs, we can define the probability that maturation is successfully finished in the absence of inhibitors $\rho_{\mathrm{PR},\phi}$ and in the presence of inhibitors $\rho_{\mathrm{PR,PI}}$

$$\rho_{\mathrm{PR},\phi} = \frac{k_{\mathrm{mat}}}{k_{\mathrm{mat}} + \mathrm{CL}} \tag{S29}$$

$$\rho_{\mathrm{PR,PI}} = \frac{(1-\varepsilon_{\mathrm{PI}}) \cdot k_{\mathrm{mat}}}{\mathrm{CL} + (1-\varepsilon_{\mathrm{PI}}) \cdot k_{\mathrm{mat}}}. \tag{S30}$$

The effect of PIs can then be defined as the decrease in the likelihood that virions will successfully maturate, before they are cleared by the immune system $(1 - \eta_{\mathrm{PI}}) = \rho_{\mathrm{PR,PI}}/\rho_{\mathrm{PR},\phi}$. We derive

$$(1 - \eta_{\mathrm{PI}}(\rho_{\mathrm{PR},\phi})) = \frac{1}{\rho_{\mathrm{PR},\phi} + \frac{1-\rho_{\mathrm{PR},\phi}}{(1-\varepsilon_{\mathrm{PI}})}}, \tag{S31}$$

analogously to Eq. (S24). Therefore in the case of PIs, we observe the same non-linearity between $(1-\eta_{\mathrm{PI}})$ and $(1-\varepsilon_{\mathrm{PI}})$ as in the case of RTIs (see Fig. S1). The effect of MIs and PIs on the production of infectious particles N can be modelled accoring to

$$\mathrm{N}_{\mathrm{PI,MI}} = (1 - \eta_{\mathrm{MI}}) \cdot \big(1 - \eta_{\mathrm{PI}}(\rho_{\mathrm{PR},\phi})\big) \cdot \mathrm{N_T}. \tag{S32}$$

Similarly, the above equation can be used to model the influence of PIs and MIs on the production of non-infectious Virus $\mathrm{V_{NI}}$.

References

1. Wei X, Ghosh SK, Taylor ME, Johnson VA, Emini EA, et al. (1995) Viral dynamics in human immunodeficiency virus type 1 infection. Nature 373: 117–122.
2. Perelson AS, Neumann AU, Markowitz M, Leonard JM, Ho DD (1996) HIV-1 dynamics in vivo: virion clearance rate, infected cell life-span, and viral generation time. Science 271: 1582–1586.
3. Perelson AS, Essunger P, Cao Y, Vesanen M, Hurley A, et al. (1997) Decay characteristics of HIV-1-infected compartments during combination therapy. Nature 387: 188–191.
4. Markowitz M, Louie M, Hurley A, Sun E, Mascio MD, et al. (2003) A novel antiviral intervention results in more accurate assessment of human immunodeficiency virus type 1 replication dynamics and T-cell decay in vivo. J Virol 77: 5037–5038.
5. Hurn AS, Lindsay KA, Michie CA (1997) Modeling the lifespan of human T lymphocyte subsets. Math Biosci 143: 91–102.
6. Sedaghat AR, Dinoso JB, Shen L, Wilke CO, Siliciano RF (2008) Decay dynamics of HIV-1 depend on the inhibited stages of the viral life cycle. Proc Natl Acad Sci U S A 105: 4832–4837.
7. Sedaghat AR, Siliciano RF, Wilke CO (2009) Constraints on the dominant mechanism for HIV viral dynamics in patients on raltegravir. Antivir Ther 14: 263–271.
8. Ross J (1995) mRNA stability in mammalian cells. Microbiol Rev 59: 423–450.
9. Houseley J, Tollervey D (2009) The many pathways of RNA degradation. Cell 136: 763–776.
10. Koelsch KK, Liu L, Haubrich R, May S, Havlir D, et al. (2008) Dynamics of total, linear nonintegrated, and integrated HIV-1 DNA in vivo and in vitro. J Infect Dis 197: 411–419.

11. Zhou Y, Zhang H, Siliciano JD, Siliciano RF (2005) Kinetics of human immunodeficiency virus type 1 decay following entry into resting CD4+ T cells. J Virol 79: 2199–2210.

12. Pierson TC, Zhou Y, Kieffer TL, Ruff CT, Buck C, et al. (2002) Molecular characterization of preintegration latency in human immunodeficiency virus type 1 infection. J Virol 76: 8518–8531.

13. Pettit SC, Lindquist JN, Kaplan AH, Swanstrom R (2005) Processing sites in the human immunodeficiency virus type 1 (HIV-1) Gag-Pro-Pol precursor are cleaved by the viral protease at different rates. Retrovirology 2: 66.

14. Perelson AS, Nelson PW (1999) Mathematical analysis of HIV-1 dynamics in vivo. SIAM Review 41: 3-44.
